# Psychosocial Factors in Diabetes and Cardiovascular Risk

**DOI:** 10.1007/s11886-016-0771-4

**Published:** 2016-08-27

**Authors:** Ruth A. Hackett, Andrew Steptoe

**Affiliations:** Department of Epidemiology and Public Health, University College London, 1-19 Torrington Place, London, WC1E 6BT UK

**Keywords:** Type 2 diabetes, Psychosocial factors, Cardiovascular disease, Depression, Stress

## Abstract

Type 2 diabetes is a chronic disease that is increasing in prevalence globally. Cardiovascular disease is a major cause of mortality and morbidity in diabetes, and lifestyle and clinical risk factors do not fully account for the link between the conditions. This article provides an overview of the evidence concerning the role of psychosocial stress factors in diabetes risk, as well as in cardiovascular complications in people with existing diabetes. Several types of psychosocial factors are discussed including depression, other types of emotional distress, exposure to stressful conditions, and personality traits. The potential behavioral and biological pathways linking psychosocial factors to diabetes are presented and implications for patient care are highlighted.

## Introduction

Diabetes is a growing problem that poses a major public health challenge globally. According to reports by the International Diabetes Federation (IDF) and the World Health Organization (WHO), over 8 % of the world population (415–420 million people) currently have diabetes, with prevalence expected to rise to 10.4 % (642 million) by 2040 [1, 2]. In the USA, an estimated 9.3 % of the population (29.1 million people) have diabetes [3]. Worldwide, type 2 diabetes is the most common form of diabetes accounting for 90 % of cases [1, 4]. Throughout this review, the term “diabetes” will be used to refer to type 2 diabetes unless indicated otherwise.

Diabetes is the fourth or fifth leading cause of mortality in most high-income countries and as such represents a significant burden to public health systems [5]. In 2015, health spending on diabetes represented 12 % (USD 673 billion) of global health expenditure and it may account for up to 20 % of national health-care budgets in some countries [1]. Additionally, the indirect costs of diabetes such as a reduced labor force and lowered economic productivity are considerable [6].

### Cardiovascular Disease in People with Diabetes

Cardiovascular disease (CVD) is a major cause of mortality and morbidity in individuals with diabetes. Results from a meta-analysis of 102 prospective studies indicate that people with diabetes have a 2-fold excess risk of CVD compared with controls. This association is independent of traditional CVD risk factors [7]. The risk associated with raised blood glucose is continuous, such that intermediate categories of glucose disturbance or “prediabetes” are associated with heightened CVD risk [7, 8]. Contemporary evidence from a longitudinal population study of 1.9 million people provides more evidence that there is a strong association between diabetes and incident CVD [9]; however, it is worth noting that the magnitude of the reported relationship varied between different CVD subtypes in this study.

In keeping with wider population time trends, CVD rates have fallen over the past decades among people with diabetes [10]. However, the reduction has been less than in the rest of the population; thus, the heightened risk of CVD in people with diabetes persists [11•, 12].

In addition to heightened CVD risk, diabetes is associated with CVD mortality. According to a review of 97 prospective studies, individuals with diabetes die 6 years earlier on average than their counterparts without the condition, and approximately 58 % of this survival difference is attributable to excess vascular deaths [13]. Similar estimates of CVD-driven excess mortality are presented in the latest IDF and WHO reports [1, 2]. In an authoritative pooled analysis of 91 longitudinal cohort studies, diabetes, stroke, and MI were found to have equivalent associations with all-cause mortality [14].

Outcomes following a cardiovascular event are substantially worse in people with diabetes. In a trial of 13,608 ST-segment elevation MI patients, participants with diabetes had significantly higher rates of recurrent nonfatal MI and cardiovascular death than patients without diabetes [15]. Interestingly, diabetes is approximately a third more strongly related to fatal than nonfatal MI [13]. Similarly, in individuals with heart failure, diabetes is an independent predictor of repeat hospitalization and cardiac death [16]. Diabetes increases the risk of recurrent stroke [17] and attenuates both cognitive and functional recovery [18]. Myocardial revascularization procedures are also challenging in individuals with diabetes; compared to patients without the condition, people with diabetes have a substantially increased risk of mortality and adverse clinical outcomes following these procedures [19, 20].

## CVD and Diabetes: A Link Beyond Traditional Risk Factors

Lifestyle factors (smoking, poor diet, physical inactivity, excess alcohol consumption), clinical factors (obesity, hypertension, raised cholesterol), and psychosocial stress have been identified as modifiable risk factors for CVD development [5, 21]. Psychosocial stress factors can be divided into negative emotional disorders (e.g., depression and anxiety), personal traits (e.g., anger or hostility), and external stressors (exposure to stressful conditions). The evidence relating sociodemographic, behavioral, and clinical risk factors with CVD risk in people with diabetes was reviewed in this journal in 2015 [22]. This article did not cover the role of psychosocial factors in diabetes and their association with CVD risk.

Furthermore, there is evidence that the link between CVD and diabetes is not fully accounted for by traditional risk factors. In meta-analyses showing greatly increased CVD morbidity and mortality in diabetes, the reported associations were robust to adjustment for clinical and behavioral risk factors [7, 13]. With regards to intervention and prevention, intensive programs targeting lifestyle factors such as diet, physical activity, and weight management have been shown to prevent diabetes onset in people with and without prediabetes [23–25]. The Diabetes Prevention Programs are recognized as some of the most effective lifestyle interventions for preventing chronic disease. Since these interventions modify CVD risk factors such high body mass index (BMI) and blood pressure [26], they in turn should have an impact on CVD outcomes. However, lifestyle interventions to reduce CVD in people with diabetes have been largely disappointing. These trial results were reviewed in 2015 in a joint report from the American Heart Association and American Diabetes Association [11•]. Evidence from prospective intervention studies such as the LookAHEAD trial [26] and the ACCORD trial [27, 28] suggests that the modification of behavioral risk factors such as weight loss, blood pressure, and cholesterol control does not significantly lower risk of adverse CVD outcomes in people with diabetes. Another survey of nearly 100 studies that looked at physical activity interventions and subsequent CVD in people with diabetes concluded that although effects have been seen in small studies, large randomized control trials (RCTs) have not found a protective effect [29]. Some positive findings regarding diet have been reported in the Spanish PREDIMED RCT. This study found that participants with diabetes who were randomized to a Mediterranean diet had a 30 % reduced risk of CVD over 4.8 years of follow-up [30]. However, these results must be interpreted with caution as a similar effect of dietary change on CVD outcomes were not reported in the LookAHEAD trial or the Diabetes Prevention Programs [23–25]. It may be that the Mediterranean diet is superior to the low-calorie diet used in other trials, but further studies are required to test this assertion.

In light of this evidence, it appears that increased risk of CVD in people with diabetes is not fully explained by traditional risk factors. Therefore, the current review focuses on psychosocial factors in diabetes. Specifically, we evaluate the role of psychosocial factors in diabetes development, as well as the evidence for the involvement of psychosocial factors translating diabetes into increased CVD risk.

## Psychosocial Factors in Cardiovascular Disease

There is accumulating evidence that psychosocial stress plays a role in the pathogenesis of CVD [31, 32, 33•]. Numerous systematic reviews and prospective analyses [34, 35] are in agreement that chronic stressors and psychosocial factors predict future coronary heart disease in initially healthy populations independently of standard risk factors. Considering the strong links between CVD and diabetes, it is not surprising that there has been interest in investigating whether psychosocial stress plays a role in diabetes and whether this is related to CVD risk in this population. The following sections review evidence from prospective studies that have examined the links between different psychosocial stress factors and diabetes.

**Fig. 1:**
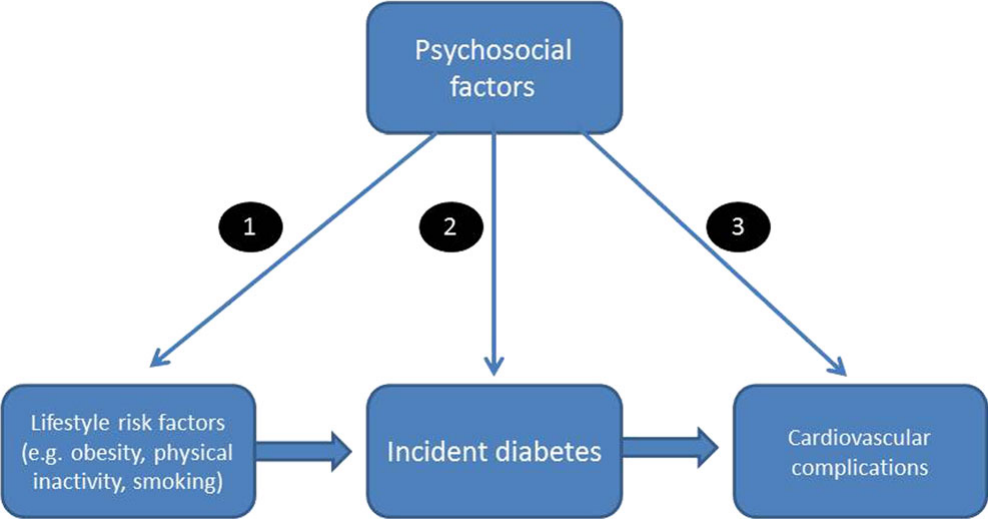
Conceptual framework

The conceptual framework in Fig. 1 indicates that psychosocial factors potentially impact on diabetes and cardiovascular risk in several ways: (1) influencing lifestyle risk factors for diabetes such as adiposity and physical activity, (2) affecting the development of diabetes directly through mechanisms such as glucose dysregulation and inflammation, and (3) shaping the processes through which diabetes stimulates cardiovascular complications. These distinctions are important both in etiological understanding and because of their implications for disease management.

## Psychosocial Factors and Diabetes Risk

### Emotional Distress

Depression is the most commonly researched factor in studies of diabetes. Results from two meta-analyses of longitudinal studies indicate that depression is associated with 37–60 % increased risk of developing diabetes [36, 37]. Prospective evidence also suggests that elevated depressive symptoms, as well as clinical depression, are related to subsequent incidence of diabetes [38, 39]. The associations reported in these studies remained significant after controlling for diabetes risk factors such as BMI, family history of diabetes, smoking, physical activity, diet, and alcohol consumption.

It should be emphasized that these studies do not prove causality, and alternative noncausal explanations are plausible [40•]. One possibility is that diabetes and depression share common etiological factors such as physical inactivity or inflammation that may not be completely eliminated by statistical adjustments. Alternatively, preclinical diabetes may increase the chances that an individual reports depression, resulting in a reverse causal process that is detailed later in this review.

Fewer studies have investigated whether anxiety is associated with diabetes development. Engum [41] investigated the association in a Norwegian population cohort of 37,291 people. Over a 10-year follow-up, individuals who reported symptoms of anxiety at baseline had an increased risk of developing diabetes (odds ratio (OR) = 1.5; 95 % confidence interval (CI) 1.3–1.8) [41]. A limitation of this study was that anxiety and depression symptoms were not investigated separately; therefore, the effect of anxiety on diabetes independent of depression could not be assessed. A similar association between anxiety alone and incident diabetes (OR = 1.6; 95 % CI 1.2–2.1) was found in the Netherlands Study of Depression and Anxiety (NESDA) [42]. However, not all longitudinal research has found an association between anxiety and subsequent diabetes [43, 44], and there may be sex differences that are poorly understood [45, 46].

Psychological distress encompasses a range of comorbid psychological factors, such as depressive and anxiety symptoms, general stress as well as sleep disturbance. In a UK study of 9514 people, psychological distress at baseline was associated with incident diabetes over 18 years of follow-up adjusting for age, sex, education, and income. However, the relationship did not remain significant after additional adjustment for health-related factors [47]. In the UK Whitehall II cohort, psychological distress did not predict diabetes in the overall sample, but in a subsample of participants at high risk of diabetes (prediabetes at baseline and >40 on the Framingham diabetes risk score), distress was associated with a 40.9 % increased risk of diabetes independent of a range of covariates [48]. No sex interaction effects were found in the UK studies, but an earlier Swedish study of 5227 individuals who were normoglycemic at baseline reported an association between distress and subsequent diabetes in male but not in female participants [49]. The reasons for the mixed findings in the studies are unclear. It may be that initial health moderates the relationship and psychological distress accelerates progression to diabetes only in high-risk individuals. Another issue is that distress in of itself may be too broad a measure and that only particular aspects of it are related to future diabetes.

### Exposure to Life Stress

Chronic exposure to external stressors has also been implicated in diabetes onset. To date, the majority of research has explored the relationship between work stress and incident diabetes [50, 51••]. Job strain, which is the combination of high job demands and low control at work, is a widely studied work stress construct [52]. A large meta-analysis investigating the link between job strain and diabetes development pooled results from 13 prospective European cohort studies. Over a mean follow-up of 10.3 years, job strain was associated with a 1.15-fold (95 % CI 1.06–1.25) increased risk of incident diabetes [51••]. This association was independent of a range of covariates and extends previous pooled cross-sectional associations [53]. A substantial number of studies of the relationship between long work hours and diabetes have also been carried out [50]. It appears that that working 55 h or more a week also increases the risk of developing diabetes but only in low socioeconomic status (SES) groups (RR 1.29, 95 % CI 1.06–1.57).

Perceived stress is a broader conceptualization of psychosocial stress exposure that has been implicated in diabetes development. Novak et al. [54] investigated the relationship between perceived permanent stress (self-reported stress related to work or home life that was ongoing for a year or more) and incident diabetes in a sample of 7251 men. Over the 35-year follow-up period, men with permanent stress had a greatly increased risk of diabetes (HR 1.52; 95 % CI 1.26–1.82) compared with those reporting no or periodic stress. This relationship was not accounted for by conventional diabetes risk factors. Similarly, Japanese men with high levels of “stress in daily life” have been found to have a greater risk of incident diabetes over a 3-year follow-up [55].

However, findings from other prospective studies with both male and female participants have been equivocal. In the Copenhagen City Heart Study involving 7066 participants, men who reported daily emotional stress were two times more likely to develop diabetes than those with low levels of stress over a 10-year follow-up period (OR = 2.36; 95 % CI 1.22–4.59), but no associations were found for women [56]. Conversely, a study of 3759 Australian men and women found no relationship between perceived stress and the development of abnormal glucose tolerance in men over 5 years of follow-up, but detected an association in women [57]. It is unclear why some studies have found sex differences, but sample size might offer one possible explanation. The largest study to date investigated the relationship between perceived stress and subsequent diabetes development in 55,826 Japanese men and women over a 10-year follow-up period [58]. In this analysis, high levels of perceived stress were found to increase the risk of diabetes onset in both men and women, but effects were stronger among male (OR 1.39; 95 % CI 1.15–1.65) than female participants (OR 1.25; 95 % CI 1.01–1.56) after adjustment for known diabetes risk factors.

Another study assessed the association between perceived stress and diabetes in 22,567 participants (71 % men) from a French workforce cohort. Over 5.3 years of follow-up, no association between perceived stress and future diabetes was observed in the full sample [59]. However, perceived stress was significantly associated with new onset diabetes in participants of low occupational status (OR 1.39; 95 % CI 1.02–1.90). The authors suggest that low occupational status might reflect a greater work stress. If this is the case, these results would map onto previous meta-analytic findings indicating that long working hours are only associated with incident diabetes in those of low SES [50].

Considering the current evidence as a whole, there appears to be an association between perceived stress and increased risk of diabetes in initially healthy populations. However, sex or SES (as measured by occupational status) may moderate this association. As in the case of depression, these longitudinal observational studies do not prove causality.

### Early Life Adversity

Early life adversity has not been widely investigated as a risk factor for future diabetes onset, though it appears to be a significant issue for health-related processes such as telomere length and inflammation in adult life [60, 61]. A review published in 2015 summarized the existing evidence on diabetes [62•], analyzing 7 prospective and cross-sectional studies with data on 87,251 participants. People who reported an adverse childhood experience had a 32 % increased odds of diabetes. Looking at different types of childhood stress, the strongest association was found for neglect (OR 1.92), followed by sexual abuse (OR 1.39) and physical abuse (OR 1.30). A limitation of this meta-analysis is that some of the studies included involved retrospective accounts of childhood adversity which may not be accurate, whereas other studies were analyses of life course data. The studies varied in diabetes measurement, with both self-reported and objective measures being used. There are unanswered questions in this area such as whether there is a critical period in exposure to early life stress or whether there is a dose-response relationship between the frequency or duration of the stress and diabetes risk.

Prenatal stress has also been suggested to play a role in the development of diabetes. In a large Danish cohort study, participants who were exposed to prenatal stress (*n* = 45,302 out of nearly 1.9 million) because their mothers experienced bereavement in their prenatal life were found to have an elevated risk of future diabetes (incidence rate ratio 1.31; 95 % CI 1.01–1.69). This association was independent of parental diabetes and other conventional diabetes risk factors [63].

### Personality Traits

Personality factors are not well researched in relation to diabetes. Hostility is a trait that is typically conceptualized as a negative cynical attitude toward others, with a propensity for anger or aggression [64]. This trait has been associated prospectively with raised fasting glucose [65] and cross-sectionally with insulin resistance [66, 67], glycated hemoglobin (HbA1c) [67, 68], and prevalent diabetes [69]. Additionally, angry temperament has been investigated in relation to diabetes development. In a cohort study of 11,615 individuals who were disease-free at baseline, individuals with an angry temperament had a 1.34 increased hazard of incident diabetes over the 6-year follow-up period [70]. This association has also been investigated in the MESA cohort [43] with a smaller sample size of 5598 participants but a longer follow-up of 11.4 years. Participants reporting high levels of trait anger at baseline had a 48 % greater risk of developing diabetes than those with low anger (HR 1.48; 95 % CI 1.04–2.12) independent of demographic factors, exercise, diet, alcohol use, and smoking. However, the association was attenuated following adjustment for waist circumference. So although there have not been many studies investigating anger and hostility, the current evidence suggests that these characteristics may also be associated with an increased risk of diabetes in later life.

### Potentially Protective Psychosocial Factors

The vast majority of work has investigated relationships between negative psychosocial stress factors and future diabetes. To our knowledge, only two studies have investigated associations with potentially protective positive factors. Crump et al. studied the relationship between resilience to stress in adolescence and diabetes in later life [71••]. The study used an impressive sample of over 1.5 million Swedish military conscripts who were assessed for stress resilience during a semistructured interview at 18 years. The participants were followed up for an average of 25.7 years using national medical records. Participants with the lowest stress resilience had a 51 % increased hazard of future diabetes compared to those with high levels of resilience (HR 1.51; 95 % CI 1.46–1.57) independent of a range of diabetes risk factors. These findings suggest that personal resilience to stress might be an important factor in the development of diabetes. The other study looked at life satisfaction, emotional vitality, and optimism in 7800 individuals from the Whitehall II cohort [72]. Over 13 years of follow-up, these well-being factors were not associated with incident diabetes as assessed by self-reported and glucose tolerance testing. The authors performed subanalyses to assess whether associations varied by type of diabetes diagnosis. Individuals with high life satisfaction (OR = 0.85; 95 % CI = 0.76–0.95) and emotional vitality (OR = 0.86; 95 % CI = 0.77–0.97) were less likely to report doctor-diagnosed diabetes. It is uncertain why associations differed for doctor-diagnosed and screen-detected diabetes.

## Psychosocial Factors and Cardiovascular Complications in People with Diabetes

The research detailed in previous sections had primarily focused on pathway 2 outlined in Fig. 1. Additionally, psychosocial factors may be relevant to the risk of cardiovascular complications in people with diabetes (pathway 3).

### Depression in Diabetes

Depression is common in people with diabetes [73–76]. A meta-analysis of 10 studies with 51,331 individuals estimated the prevalence of depression to be almost doubled in people with diabetes compared to those without the condition (17.6 vs. 9.8 %; OR = 1.6; 95 % CI 1.2–2.0) [75]. The vast majority of the studies have been conducted in Western countries, but a study of 213,797 people in 47 countries from around the world has shown that people with diabetes had a 2-fold greater prevalence of depressive symptoms than those without diabetes (OR 2.36; 95 % CI 1.91–2.92) [73].

As well as increased prevalence in people with diabetes, longitudinal studies indicate that diabetes diagnosis is a risk factor for incident depression [37, 77, 78]. An analysis pooling data from 16 longitudinal studies involving 497,223 participants with an average follow-up of 5.8 years indicated that people with diabetes have a 25 % increased risk of developing depression compared with controls without diabetes [78]. More recent longitudinal studies have confirmed this relationship. For example, an analysis of the English Longitudinal Study of Aging (ELSA) cohort found that diabetes was associated with a doubling in the odds of depressive symptoms over 4 years of follow-up [42], while a larger study of 37,043 Swedish twins with diabetes showed 33 % increased risk of major depression [41]. However, in both of these studies, the association was only found in younger participants with diabetes (≤55 or 64) and not in older individuals. The reasons are not known. It might be that middle-aged people with diabetes perceive their condition as a disease of old age that should not have happened to them and become more despondent following diagnosis. Alternatively, it could be that younger patients have more competing priorities (e.g., employment, raising children, and financial commitments such as mortgages) and find diabetes care a greater burden to manage in comparison with older patients who are likely to have a grown-up family and be retired. There is some evidence from smaller cross-sectional studies that diabetes management might be more challenging for younger than older adults [79, 80] but stronger prospective evidence from larger samples is required to explore these possibilities further.

It should be pointed out that the link between depression and diabetes is not unique to this condition. Similar associations have been observed for other common diseases such as osteoarthritis, coronary heart disease, chronic lung diseases, and stroke [81, 82]. Nevertheless, comorbid depression in diabetes is a considerable threat to quality of life in people with diabetes [83, 84] and is of clinical importance since it has been associated with suboptimal glycemic control [85, 86] and nonadherence to treatment regimens [87, 88], as well as with the microvascular [89, 90] and macrovascular complications of diabetes. This review focuses on the risk of macrovascular complications.

### Depression and Complications

An increasing body of literature indicates that depression exacerbates the risk of macrovascular complications in people with diabetes. Prospective studies have found a relationship between comorbid depression and CVD in diabetes samples [91, 92]. For instance, in the Pathways Epidemiological Follow-up Study of over 4000 participants with diabetes, individuals with depression had a 24 % increased risk of adverse macrovascular complications including MI, stroke, cardiovascular procedures, and cardiac death over 5 years of follow-up [92]. This association was independent of prior complications, sociodemographic characteristics, health behaviors, and diabetes self-care variables. Another study followed a cohort of 345,949 individuals who were free of CVD at baseline over a 7-year period. Results showed that participants with diabetes alone and those with major depression alone had a 30 % increased risk of MI, whereas those with a double diagnosis (both diabetes and major depression) had an 82 % excess risk of subsequent MI compared to participants without either condition [93].

Another study published in 2016 examined the association between depressive symptoms or perceived stress at baseline and risk of CVD in 22,003 US adults [94]. Over almost 6 years of follow-up, people with diabetes who reported elevated depressive symptoms or perceived stress had a significantly increased incidence of stroke (HR 1.57; 95 % CI 1.05–2.33) and acute CVD (HR 1.57; 95 % CI 1.02–2.40). These associations were independent of demographics factors but were attenuated when controlling for lifestyle factors. The majority of studies have focused on white samples in Western countries. To address this issue, Ting et al. [95•] conducted a 7.4-year follow-up study in a sample of 7835 Chinese patients with diabetes who were free of CVD at baseline. In this sample, diagnosed depression predicted CVD [HR = 2.18] and the majority of the risk appeared to be driven by stroke [HR = 3.55]. The reported associations remained significant after adjustment for a wide range of conventional CVD risk factors.

As might be expected from these findings, people with both depression and diabetes have greater CVD mortality as well as morbidity rates. An analysis of 16 prospective studies indicated that comorbid depression in diabetes is associated with a 39 % increased risk of cardiovascular death and a 46 % higher risk of all-cause mortality [97].

### Anxiety in Diabetes

In comparison with the research on comorbid depression, there is relatively little research on other psychosocial factors and their relationship to CVD risk in people with diabetes. However, there is some evidence that the prevalence of other psychosocial disorders is elevated in people with diabetes in comparison with the general population. Overviews of this research suggest that diabetes is associated with 20 % increased odds of having an anxiety disorder and 48 % increased odds of having elevated anxiety symptoms [96]. Comorbid anxiety is of clinical relevance to people with diabetes. An association between anxiety and glycemic control has also been reported in a number of studies [97], but to our knowledge, only one study to date has investigated the prospective relationship between anxiety and diabetes complications such as retinopathy, neuropathy, or CVD, and no associations were found [44]. However, the number of cases of diabetes and anxiety was limited in this study, so it may have been unpowered to detect such effects.

### Psychological Distress and Diabetes-Specific Distress

Psychological distress has been linked with CVD morbidity and increased mortality rates in people with diabetes. In a study of 1533 individuals with diagnosed diabetes, distressed participants were found to have a 1.69 increased hazard of a CVD event and a 1.76-fold greater mortality rate compared to individuals without psychological distress over an average follow-up of 5.4 years [98]. This association was independent of CVD risk factors, and excluding participants who were receiving antidepressive treatment did not impact the results. It would be interesting in future studies to tease out what particular aspects of psychological distress are most strongly linked to CVD.

As well as depression, anxiety, and general psychological distress, diabetes-related emotional distress, a stress condition specifically resulting from concerns and worries about diabetes and its management, is common in people with diabetes [99]. According to estimates from a study of 8596 adults with diabetes from 17 countries, 44.6 % of patients suffer from significant diabetes-related distress [100]. Diabetes-related distress is of clinical significance, since several longitudinal studies suggest that it is associated with poor glycemic control independently of depression [99, 101, 102]. No studies to date have investigated the association between diabetes-distress and macrovascular complications.

## Pathways Linking Psychosocial Factors and Diabetes

The precise mechanisms linking psychosocial factors and diabetes remain to be elucidated. However, several potentially interrelated pathways that plausibly account for the link have been proposed. As noted in Fig. 1, one possibility is that the adverse relationship between psychosocial factors and diabetes may be mediated via behavioral pathways. Behavioral mechanisms include poor diet, physical inactivity, excess alcohol consumption, and smoking. Reduced adherence to self-care behaviors and cardioprotective medications such as blood pressure and lipid-lowering drugs could also play a role.

Several lines of research support this mechanism. Comorbid depression in diabetes increases nonadherence to a range of behaviors including diet, medication usage, and exercise [87, 88]. For example, a study of 2759 individuals with diabetes found that participants with persistent or increasing depression symptoms had significantly poorer adherence to dietary and exercise regimens than their counterparts without depression over a 5-year follow-up period [88]. A cross-sectional study with a similar sample size of 2646 individuals with diabetes reported that physical inactivity doubled in the presence of depressive symptoms [103]. Considerably less research has investigated the behavioral pathways linking other psychosocial factors and diabetes. The Copenhagen City Heart Study of 7066 adults found that perceived stress was associated with physical inactivity and unsuccessful smoking cessation or alcohol reduction attempts over a 10-year follow-up as well as the development of overt diabetes among men [56].

Negative psychosocial factors that are common in diabetes may also decrease motivation for healthy lifestyle choices that in turn impact CVD risk. However, the association between diabetes and CVD is not fully explained by behavioral risk factors [7, 13], and results from RCTs suggest that the modification of behavioral risk factors does not significantly lower CVD outcomes in people with diabetes, despite the fact that lifestyle change has a marked effect on diabetes incidence [11•]. This offers the possibility that a direct biological pathway could link psychosocial factors with CVD risk in people with diabetes.

Several different psychobiological mechanisms could link psychosocial factors with both CVD and diabetes. Depression, anxiety, and diabetes-related distress are associated with suboptimal glycemic control [85, 86, 97, 102, 104], and there is evidence that the risk of CVD increases in line with the degree of hyperglycemia [7, 105]. Long-term exposure to psychosocial stress factors can result in chronic allostatic load which involves dysregulation of multiple biological systems including neuroendocrine, cardiovascular, metabolic, and inflammatory pathways [106]. Cross-sectional evidence suggests that people with diabetes demonstrate characteristics of high allostatic load in response to a stressful experience [107•]. Evidence from the same sample suggests that hostility (a psychosocial stress factor) can further exaggerate the disturbances in stress-related processes in people with diabetes [108].

Many of the different pathways involved in stress-related allostatic load have been linked with both diabetes and CVD. Dysregulation of the stress hormone cortisol is associated with both acute and chronic stress factors [109], and prospective evidence has linked changes in daily cortisol secretion with CVD mortality [110], as well as new onset diabetes [111]. Another potential biological mechanism linking psychosocial factors to both CVD and diabetes is inflammation. Elevated inflammatory cytokine concentrations are observed in people reporting high levels of psychosocial stress [112]. Both diabetes [113] and CVD have been characterized as inflammatory conditions [114]. In initially healthy populations, heightened inflammatory cytokines levels have been associated with new onset CVD [115], and it is plausible that raised cytokine levels could increase the risk of CVD in diabetes populations, too.

The complexity of the link between psychosocial factors and CVD risk in people with diabetes is reflected in the numerous pathways that may be involved. It is likely that the proposed behavioral and biological pathways do not act in isolation but rather that they are interrelated. For example, depression in diabetes might impact complications through poorer adherence to diet and exercise regimes. Future research in this area may benefit from investigating the synergies between behavioral and biological pathways in linking psychosocial factors to CVD risk in people with diabetes.

## Implications for Patient Care

Lifestyle interventions to prevent diabetes have been successful [23–26]. However, interventions to modify behavioral risk factors to prevent CVD in people with diabetes have been largely disappointing. This poses the question whether modifying psychosocial stress, another risk factor for CVD, would have a therapeutic impact.

To date, the majority of research in this area has investigated whether the treatment of depression in diabetes is beneficial. A Cochrane review in 2012 included 19 RCTs investigating both psychological and pharmacological interventions for depression in patients with diabetes [116]. Psychological intervention studies showed a beneficial effect on short-, medium-, and long-term depression severity and had a good impact on depression remission compared to usual care. However, the effect of psychological intervention on glycemic control was mixed and inconclusive. With regards to the pharmacological interventions, there was a moderate effect of antidepressant medication on short-term depression severity and depression remission, and interestingly, the pharmacological trials significantly improved glycemic control in the short term as well. But no study to date has assessed the relationship between depression treatment and glycemic control in the longer term. Taking the evidence together, it appears that depression treatment is moderately effective in diabetes, but only pharmacological trials have shown a consistent improvement in glycemic control.

Mindfulness-based interventions for modifying psychosocial stress factors have also been tested in people with diabetes [117•]. They have been found in several studies to have psychological benefits, lowering depression, anxiety stress, and diabetes-distress symptoms in people with diabetes. However, the evidence for the effectiveness of these interventions on glycemic control is mixed. Out of the seven studies that assessed HbA1c as a marker of glycemic control, four interventions lowered HbA1c levels, but the three largest studies reported no change in HbA1c [117•]. Mindfulness-based intervention in diabetes is a new field, and much of the research is exploratory. It may be that the short follow-up periods of many studies were not sufficient to observe significant changes in HbA1c.

There is limited evidence that the treatment of psychosocial factors can reduce the risk of adverse outcomes in people with diabetes [118]. The Prevention of Suicide in Primary Care Elderly Collaborative Trial (PROSPECT) RCT was used to investigate whether depression management would decrease mortality in diabetes [119]. Depressed people with diabetes who were assigned to the intervention group (an individualized case management approach) had significantly lower mortality rates than controls over a 5-year follow-up (HR 0.49; 95 % CI 0.24–0.98). However, this study has been criticized with regards to study design and analysis, with suggestions that the methods may not have been appropriate [120]. The impact of mindfulness interventions on CVD outcomes in people with diabetes has not yet been examined [117•].

In sum, there is little evidence as yet that the treatment of psychosocial factors in diabetes has a benefit on CVD outcomes. However, pharmacological interventions have been shown to improve glycemic outcomes in the short term and hyperglycemia is linearly associated with increased CVD risk [7, 105]. Additionally, both psychological and pharmacological treatments as well as mindfulness-based interventions appear to have beneficial effects on psychosocial factors in people with diabetes. Despite the limited effectiveness of these treatments on overt CVD outcomes, there have been calls that the psychological well-being in people with diabetes should be a priority for its own sake [121]. Since lifestyle interventions have been shown to be effective for preventing diabetes, perhaps targeting individuals before onset rather than after diagnosis might be the optimal strategy.

## Conclusion

There is an emerging body of evidence that psychological stress factors play a role in the pathogenesis of diabetes. A variety of negative psychosocial factors have (for the most part) been shown to increase the risk of diabetes in initially healthy populations. There is less research on the involvement of psychosocial factors in CVD risk in people with existing diabetes. To date, most of the research in this area has been on depression, with evidence that a double diagnosis of diabetes and depression increases the risk of CVD in this population. Most of the studies are observational, so causal conclusions are difficult to draw.

The mechanisms through which psychosocial stress factors increase diabetes risk and affect outcomes in people with existing diabetes are yet to be fully understood. It is likely that both behavioral and biological pathways are involved. Interventions have been shown to have a beneficial effect on psychosocial factors in people with diabetes, but the evidence for effects on glycemic control is mixed, and research on interventions impacting CVD outcomes in people with diabetes is lacking. Despite the limited evidence for effects on physiological outcomes, it can be argued that improving the psychological well-being of people with diabetes should be a priority in its own right.
